# Account of Diseases in an Eastern District of London, from December 20, 1807, to January 20, 1808

**Published:** 1808-02

**Authors:** 


### *Account of Diseases in an Eastern District of London, from December* 20, 1807, *to January* 20, 1808.

| | |
|---|---|
| **ACUTE DISEASES.** | |
| Pneumonia | 3 |
| Peripneumonia Notha | 5 |
| Ischuria | 1 |
| Rubeola | 2 |
| **CHRONIC DISEASES.** | |
| Tussis | 5 |
| Dyspnœa | 9 |
| Tussis cum Dyspnœa | 15 |
| Catarrhus | 3 |
| Phthisis Pulmonalis | 2 |
| Pleurodyne | 3 |
| Hæmoptysis | 2 |
| Hydrothorax | 3 |
| Anasarca | 4 |
| Hemiplegia | 2 |
| Paralysis | 1 |
| Convulsio | 1 |
| Palpitatio Cordis | 2 |
| Dysuria | 4 |
| Amenorrhœa | 3 |
| Menorrhagia difficilis | 2 |
| Rheumatismus Chronicus | 14 |
| **PUERPERAL DISEASES.** | |
| Ephemera | 6 |
| Peritonitis | 1 |
| Dolores post partum | 5 |
| Menorrhagia Lochialis | 4 |
| Rhagas papillæ | 3 |
| **INFANTILE DISEASES.** | |
| Icteritia | 1 |
| Vermes | 2 |
| Ophthalmia | 2 |
| Dentitio | 3 |

Icteritia, mentioned in the list of Infantile Diseases, is of so rare occurrence, that but little notice has been taken of it by writers. The red gum, or rather the white or yellow gum, has by some been considered a sort of infantile jaundice; but the disease now referred to is very different from that. Instead of appearing in distinct patches, Icteritia covers the whole surface of the body with a deep yellow tinge. The tunica conjunctiva of the eye is also affected in the same manner; though, in the case referred to in the list, it was much less affected than in the case of adults. The urine was sufficiently impregnated to produce a deep yellow stain on the linen. This disease may be attributed to some obstruction in the biliary ducts, or to some viscid matter impeding the flow of bile into the duodenum. It has been conjectured, that in some instances the disease might be communicated from the mother to the infant at her breast; in the present instance, however, there was no appearance of disease in the mother. With respect to the mode of cure, it may be observed, that gentle and repeated aperients, and emetics have been generally found to be the most efficacious remedies in this disease.

INTELLIGENCE.

